# P-1297. Screening and vaccination for tetanus, hepatitis A, and hepatitis B in individuals with substance use disorders, hospitalized at a U.S. integrated healthcare system

**DOI:** 10.1093/ofid/ofae631.1478

**Published:** 2025-01-29

**Authors:** Jennifer H Ku, Yuqian M Gu, Cheyne Hoke, Tsai Yu Tseng, Yi Luo, Rulin C Hechter, Bradley Ackerson, Cara D Varley

**Affiliations:** Kaiser Permanente Southern California, Pasadena, California; Kaiser Permanente Southern California, Pasadena, California; Kaiser Permanente Southern California, Pasadena, California; Kaiser Permanente Southern California, Pasadena, California; Kaiser Permanente Southern California, Pasadena, California; Kaiser Permanente Southern California Department of Research and Evaluation, Los Angeles, California; Kaiser Permanente Southern California, Pasadena, California; Oregon Health & Science University, Portland, OR

## Abstract

**Background:**

Hepatitis A (HAV), hepatitis B virus (HBV), and tetanus are common vaccine preventable diseases among individuals with substance use disorders (SUDs). Inpatient encounters are frequent for this population thus represent an important opportunity for preventive care. However, up-to-date data on screening and vaccination for this high-risk population are lacking in the inpatient setting.

**Table 1.**
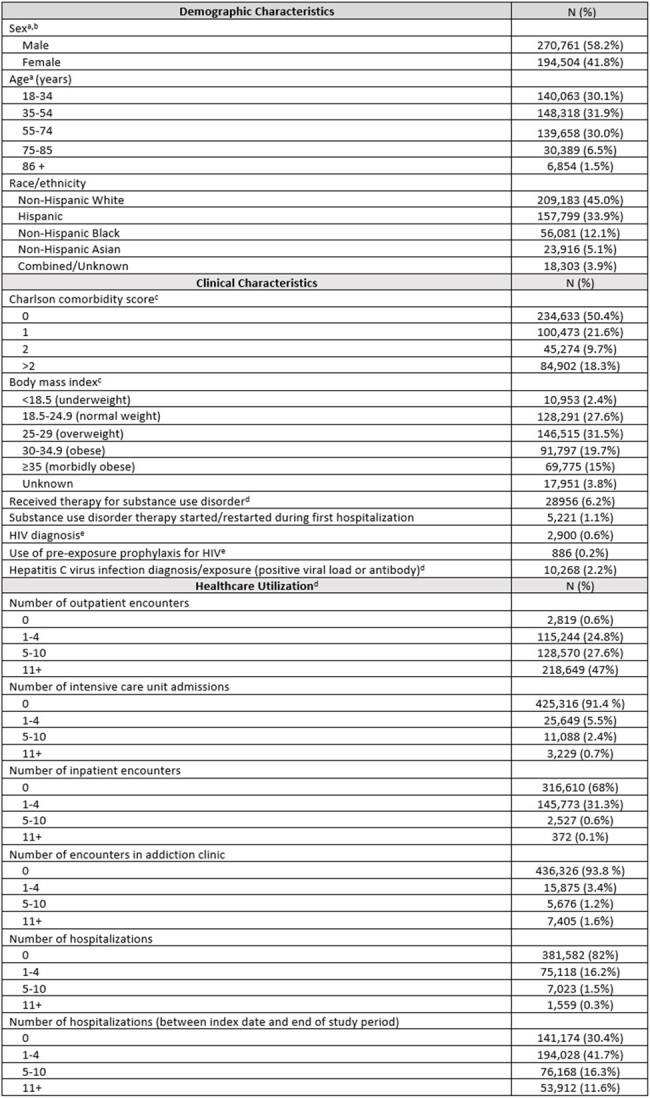
Characteristics of 465,282 individuals hospitalized at Kaiser Permanente Southern California between 2010 and 2023 with a history of substance use disorder a At the time of index date b Does not include 17 individuals identified as "Other" sex c In the 24 months prior to index date d In the 12 months prior to index date e Any time prior to index date

**Methods:**

Using electronic health records at Kaiser Permanente Southern California (KPSC), we included adults hospitalized between 01/2010 and 06/2023 (study period), with a diagnosis code for SUDs within 2 years prior to index date (first hospitalization admission date during study period). We identified those at risk for HAV and HBV at index date by labs, and evaluated vaccination during any hospitalization or within 90 days of hospital discharge (surveillance period) during study period for tetanus, HAV and HBV.

**Table 2.**
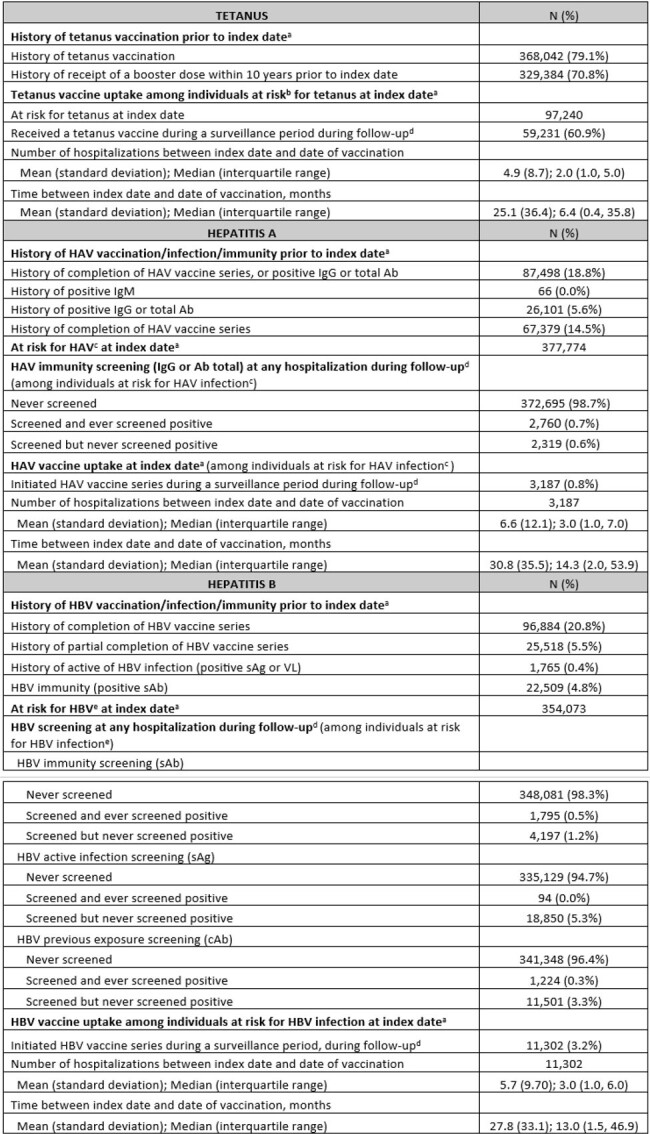
Screening and vaccination for tetanus, hepatitis A, and hepatitis B among 465,282 individuals hospitalized at Kaiser Permanente Southern California with a history of substance use disorder between 2010 and 2023 a Index date is defined as the date of the first hospital admission (including observation visits lasting >24 hours and emergency department visits) during the study period, with a diagnosis code for substance use disorder within 2 years prior b “At-risk for tetanus” is defined as no history of tetanus vaccination within 10 years prior to index date and no history of booster dose if the initial vaccination was completed >10 years prior to index date c “At-risk for HAV infection” is defined as no history of ≥2 dose of HAV vaccination, no history of positive test result for HAV IgG, HAV total Ab, and no history of HAV IgM prior to index date d Follow-up is defined as time period from the index date to the end of KPSC membership, the end of study period (i.e., June 30, 2023), or death, whichever comes first e “At risk for HBV infection” is defined as no history of ≥3 dose of HBV vaccination, no history of positive test result for HBV sAb, and no active of HBV infection (HBV viral load, or HBV sAg) prior to index date

**Results:**

We identified 465,282 individuals meeting the study criteria (42% female, 45% non-Hispanic white, mean age 48 years [SD 19]) (**Table 1**). For tetanus, 79% completed ≥1 dose prior to index date, and 71% received a booster within 10 years prior to index date (**Table 2**). Among those at risk for tetanus at index date, 61% were vaccinated during a surveillance period, after median 2 (IQR 1, 5) hospitalizations since index date. For HAV, 15% completed vaccination prior to index date. Among those at risk for HAV, 2% were screened for immunity at any hospitalization during follow-up; 1% were vaccinated during a surveillance period, after median 3 (IQR 1, 7) hospitalizations since index date. For HBV, 21% completed and 6% partially completed vaccine series prior to index date. Among those at risk for HBV at index date, 2% and 5% were screened for immunity and active infection, respectively, at any hospitalization during follow-up; 3.2% were vaccinated during a surveillance period, after median 3 (IQR 1, 6) hospitalizations since index date.

**Conclusion:**

Few with SUDs were screened or vaccinated for tetanus, HAV, or HBV during a hospitalization or at subsequent visits, after a median 2 - 3 hospitalizations after index date. This represents an opportunity for improvement in preventive care and vaccination for this high-risk population in the inpatient setting.

**Disclosures:**

**Jennifer H. Ku, PhD MPH**, GSK: Grant/Research Support|Moderna: Grant/Research Support **Yuqian M. Gu, MS**, GSK: Grant/Research Support **Yi Luo, PhD**, GlaxoSmithKline: Grant/Research Support|Moderna: Grant/Research Support|Pfizer: Grant/Research Support **Rulin C. Hechter, MD, PhD, MS**, Gilead Sciences: Grant/Research Support **Bradley Ackerson, MD**, Dynavax: Grant/Research Support|GlaxoSmithKline: Grant/Research Support|Moderna: Grant/Research Support|Pfizer: Grant/Research Support

